# Identification of novel hub genes and pathways predictive of fibrosis progression in cancer-related lymphedema through integrated multi-omics

**DOI:** 10.3389/fimmu.2025.1625972

**Published:** 2025-08-13

**Authors:** Junzhe Chen, Yuezhong Chen, Liangliang Wang, Yaping Deng, Yan Zhou, Yun Wang, Shune Xiao, Chengliang Deng

**Affiliations:** ^1^ Department of Burns and Plastic Surgery, Affiliated Hospital of Zunyi Medical University, Zunyi, Guizhou, China; ^2^ The Collaborative Innovation Center of Tissue Damage Repair and Regeneration Medicine, Zunyi Medical University, Zunyi, Guizhou, China

**Keywords:** cancer-related lymphedema, fibrosis, bioinformatics analysis, inflammation, asporin

## Abstract

**Background:**

Cancer-related lymphedema (CRL) is a common chronic complication following cancer treatment, characterized by impaired lymphatic drainage, interstitial fluid retention, and progressive fibrosis. Although the mechanisms of hypertrophic scar (HTS) fibrosis have been extensively investigated, the molecular drivers of fibrosis in CRL remain unclear. Identification of reliable biomarkers and novel therapeutic targets is essential for enabling early intervention.

**Methods:**

Transcriptomic datasets of CRL and HTS from the Gene Expression Omnibus (GEO) were integrated to identify fibrosis-associated differentially expressed genes (DEGs) and construct co-expression modules. Weighted gene co-expression network analysis (WGCNA) and protein–protein interaction (PPI) network analysis were employed to nominate hub genes. Single-cell RNA sequencing (scRNA-seq) data were used to localize candidate gene expression within immune and mesenchymal cell populations. The most promising biomarker was validated in clinical CRL tissues by Masson’s trichrome staining and Western blotting, and Pearson correlation analyses were performed to assess its association with collagen deposition and disease duration.

**Results:**

A total of 154 fibrosis-related genes were found to be shared by CRL and HTS. Among them, Asporin (ASPN) emerged as the most promising hub gene, with markedly elevated expression in late-stage CRL tissues. scRNA-seq analysis revealed that adipose-derived stem cells (ADSCs) were the predominant ASPN-expressing population. In CRL lesions, ASPN expression levels showed significant positive correlations with disease duration, TGF-β expression, and collagen accumulation.

**Conclusions:**

ASPN is identified as a key molecular biomarker of fibrosis in CRL. Its predominant expression in ADSCs and strong association with progressive tissue remodeling suggest that ASPN holds potential as both a diagnostic indicator and a therapeutic target for CRL-related fibrosis.

## 1 Introduction

Cancer-related lymphedema (CRL) is a chronic pathological condition characterized by impaired lymphatic drainage following oncologic treatments, including surgery, radiotherapy, and chemotherapy (1). With the global incidence of cancer on the rise, CRL has become one of the most common forms of lymphedema, especially in the upper limbs after breast cancer surgery (8.4% to 21.4% prevalence) and in the lower limbs following gynecological procedures (20% to 60% prevalence) (2, 3). The pathophysiology of lymphedema involves more than just fluid accumulation; it triggers a cascade of inflammation, fibrosis, and adipose tissue deposition in the affected limb (4–6). In advanced stages, the lymphedematous tissue undergoes fibroadipose remodeling – a combination of fibrosis formation and fat accumulation – which results in chronic swelling, skin changes, and functional impairment that significantly reduce patients’ quality of life(QOL) (7).

A predominant limitation in current research on CRL-related fibrosis lies in the disproportionate emphasis on fibroblasts. Recent studies, however, have demonstrated that the CRL microenvironment is enriched with profibrotic cytokines such as IL-4, IL-13, and TGF-β. These factors drive fibroblast activation and extracellular matrix(ECM) deposition, thereby accelerating fibrotic progression (8–10). Recent studies have highlighted that adipose tissue expansion is a central feature of lymphedema pathology (11). Substantial evidence indicates that the increased limb volume in chronic lymphedema is largely due to excessive adipose tissue deposition rather than just residual fluid. Particularly in subcutaneous regions where fibrosis is most severe in CRL, adipose tissue harbors a rich network of lymphatic vessels. Inflammation and fibrosis within this tissue can disrupt lymphangiogenesis and impair lymphatic drainage, ultimately exacerbating lymphedema and leading to irreversible limb swelling. However, despite the prominence of adipose fibrosis in CRL, this component remains underexplored, and the contribution of adipose-derived mesenchymal stem cells (ADSCs) has yet to receive adequate attention (1, 12).

Hypertrophic scars (HTS) represent a prototypical fibroproliferative disorder marked by persistent inflammation, aberrant wound healing, and ECM deposition. These pathological features arise from sustained mesenchymal cell activation and profibrotic signaling cascades, particularly involving TGF-β, IL-6, and Wnt pathways. Notably, HTS shares substantial mechanistic overlap with CRL-associated fibrosis, including chronic inflammatory milieu and excessive collagen production. Leveraging HTS as a comparative model thus provides a valuable opportunity to uncover core molecular drivers underpinning fibrotic remodeling. In the present study, we incorporated transcriptomic data from HTS tissues to refine and validate fibrosis-related gene signatures identified in CRL. This integrative strategy facilitated the identification of conserved fibrotic pathways and robust biomarkers with potential translational significance across diverse fibrotic disorders. Moreover, although CRL fibrosis shares common pathological features with other fibroproliferative disorders such as HTS—including chronic inflammation, mesenchymal cell activation, and excessive ECM deposition—the shared molecular mechanisms across fibrotic diseases, particularly in the context of lymphedema, remain poorly understood (13, 14). Emerging evidence suggests that ADSCs, beyond their regenerative potential, play a pivotal role in orchestrating local immune responses and fibrosis within the adipose microenvironment. These cells contribute to the maintenance of the inflammatory milieu and promote fibrotic remodeling (15, 16). However, the precise role of ADSCs in CRL-associated fibrosis has not yet been systematically investigated. There is, therefore, an urgent need to better understand adipose fibrosis in CRL and to identify key molecular players that could serve as targets for intervention or markers for disease progression.

In the present study, we addressed this gap by applying an integrative multi-omics and machine learning framework to investigate the molecular mechanisms underlying adipose tissue fibrosis in CRL. By leveraging transcriptomic data from both CRL and HTS tissues, alongside single-cell and protein-level analyses, we aimed to identify shared fibrotic pathways and discover novel biomarkers or therapeutic targets associated with fibrosis progression in CRL.

## 2 Methods

### Study design and data sources

**Figure 1:**
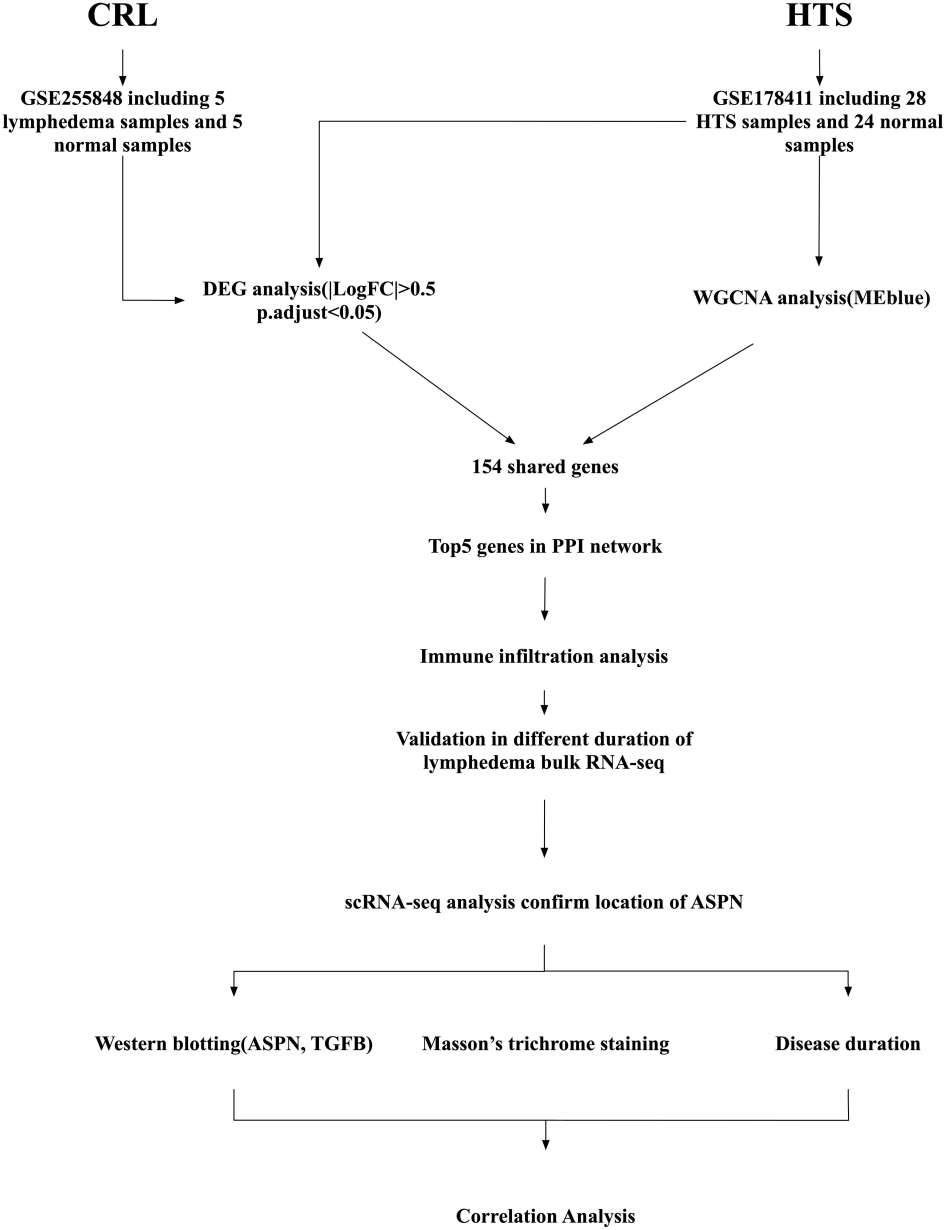
Schematic diagram of the study workflow. Transcriptomic data were collected from two public datasets: GSE255848, which includes 5 subcutaneous adipose tissue samples from patients with cancer-related lymphedema (CRL) and 5 matched normal samples; and GSE178411, which includes 28 hypertrophic scars(HTS) samples and 24 normal skin samples for HTS. Differentially expressed gene (DEG) analysis was performed on the CRL and HTS dataset, while weighted gene co-expression network analysis (WGCNA) was conducted on the HTS dataset to identify the most relevant module (MEblue). A total of 154 shared fibrosis-related genes were identified by overlapping results from DEG and WGCNA analyses. A protein–protein interaction (PPI) network was constructed, and the top five hub genes were identified based on network connectivity. These were further analyzed for immune cell infiltration profiles and validated in a bulk RNA-sequencing dataset comparing short- and long-duration CRL. Single-cell RNA sequencing (scRNA-seq) data were then used to determine the cellular localization of Asporin (ASPN). Finally, experimental validation included western blotting for ASPN and transforming growth factor beta (TGFB1), Masson’s trichrome staining for collagen deposition, and correlation analysis with disease duration to confirm clinical relevance.

A multi-step integrative bioinformatics and experimental validation approach was employed (**Figure 1**).Transcriptomic data related to CRL and HTS were obtained from the GEO database (https://www.ncbi.nlm.nih.gov/geo/), including multiple datasets used for subsequent analysis.The CRL dataset (GSE255848) comprised five subcutaneous adipose tissue samples from CRL patients and five matched healthy controls. The HTS dataset (GSE178411) included 108 samples. For this study, we selected 28 HTS samples and 24 normal controls after excluding non-relevant samples, such as wound healing samples, which were not pertinent to the fibrotic processes under investigation. To validate candidate biomarkers, two additional datasets were analyzed: (1) a scRNA-seq dataset of stromal vascular fraction (SVF) cells from CRL and healthy donors (GSA: HRA000901) (https://ngdc.cncb.ac.cn/gsa-human/browse/HRA000901) (15); and (2) a bulk RNA-seq dataset containing 10 CRL and 10 control skin samples, further stratified by disease duration between short-term (<3 years) and long-term (>14 years) (17).

### Differential expression analysis

Differentially expressed genes (DEGs) were independently identified for the CRL (GSE255848) and HTS (GSE178411) datasets using the DESeq2 package in R (v4.4.2). Thresholds were set at adjusted p < 0.05 and |log2FC| > 0.5. DEGs were visualized using volcano plots via the ggvolcano package. Shared DEGs between the two conditions were identified using a Venn diagram tool (https://bioinfogp.cnb.csic.es/tools/venny/index.html).

### Weighted gene co-expression network analysis

WGCNA was performed exclusively on the HTS dataset (GSE178411), which includes 52 samples. The CRL dataset (GSE255848) was not suitable for network construction due to its small sample size (n = 10), which is below the generally recommended minimum for robust module detection. The top 25% most variable genes were selected, and a scale-free topology was achieved with soft-threshold power β = 22 and R² = 0.85. Modules with similar expression profiles were merged using a cut height threshold of 0.25, with the minimum module size set to 30 genes. Modules significantly associated with disease phenotype were retained for cross-validation with DEG results.

### Protein-Protein Interaction(PPI) network analysis

Shared DEGs(CRL and HTS) and WGCNA(HTS) were mapped to the STRING database (confidence score > 0.4), and the resulting protein–protein interaction (PPI) network was visualized using Cytoscape ((http://www.cytoscape.org). The top five hub genes were selected based on node degree and connectivity.

### Exploratory machine learning analysis

To support and cross-validate findings from the PPI network analysis, an exploratory machine learning analysis was performed using two algorithms—Random Forest (RF) and Least Absolute Shrinkage and Selection Operator (LASSO). These analyses were conducted on the top five hub genes identified from the PPI network, rather than the full transcriptome, to reduce dimensionality and mitigate overfitting due to limited sample size. RF was implemented using the randomForest package, and LASSO regression via the glmnet package in R. Genes consistently selected by both algorithms were considered supportive fibrosis-associated candidates and are reported in the **Supplementary Material** for reference.

### Immune cell infiltration analysis

The xCell algorithm was used to estimate the relative abundance of 64 immune and stromal cell types based on bulk RNA-seq data. Immune profiles of CRL and HTS samples were visualized with ggplot2 to reveal immune microenvironmental alterations.

### Functional enrichment analysis

Functional enrichment analysis was conducted on CRL-associated DEGs, with particular focus on genes expressed in ADSCs. Gene Ontology (GO) and Kyoto Encyclopedia of Genes and Genomes (KEGG) enrichment analyses were conducted using the clusterProfiler package, with adjusted p-values < 0.05 considered statistically significant (18, 19). Enrichment results were displayed via lollipop plots.

### Venn diagram

All Venn diagrams in this article were generated using an online tool.(https://bioinfogp.cnb.csic.es/tools/venny/index.html).

### Single-cell transcriptomic and cell communication analysis

scRNA-seq data (HRA000901) were processed using Seurat (v4.4.0) and Loupe Browser 8.

Quality control steps included excluding cells with >20% mitochondrial gene content, <500 detected genes, <1,000 UMI counts, or >6,000 genes (to avoid doublets). We applied DoubletFinder (v2.0.3) to identify and remove potential doublets, estimating doublet rates based on the number of loaded cells. Cells with >50% ribosomal gene content were also excluded. To mitigate ambient RNA contamination, we used SoupX (v1.6.2) with default parameters prior to data normalization. After QC, the top 3000 most variable genes were normalized using variance-stabilizing transformation, followed by PCA, clustering (FindNeighbors, FindClusters), and UMAP visualization. Gene expression was explored using FeaturePlot. Intercellular communication networks were inferred with CellChat (v1.5.0), focusing on ligand–receptor interactions relevant to fibrosis and inflammation. AUCell (v1.22.0) was used to assign fibrosis and inflammatory scores to each single cell based on curated gene sets.

### Clinical sample collection and ethics

A total of 11 female patients diagnosed with CRL were recruited from the Department of Burns and Plastic Surgery, Affiliated Hospital of Zunyi Medical University between January 1, 2024, and January 1, 2025, for validation. Inclusion criteria were: age between 35 and 70 years, confirmed stage II–III lower limb CRL, disease duration of at least 2 years, and no concurrent systemic fibrotic or autoimmune conditions. All patients were in the chronic phase of CRL-associated fibrosis. This study was approved by the Institutional Ethics Committee of the Affiliated Hospital of Zunyi Medical University, and written informed consent was obtained from all participants. Tissue samples were collected as discarded material during clinically indicated surgical procedures. No additional intervention beyond routine care was performed. Ethical principles and patient autonomy were fully respected during recruitment.

### Masson’s trichrome staining

Paraffin-embedded tissue sections (4 μm thick) were stained with Masson’s trichrome to evaluate collagen deposition and fibrotic remodeling. Sections were imaged using bright-field microscopy. For each sample, three representative high-power fields were selected, and the percentage of collagen fibers relative to the total tissue area was calculated and averaged.

### Western blot analysis

Human skin tissue samples were collected from consenting individuals. Total proteins were extracted using RIPA lysis buffer and quantified via the BCA assay. Equal amounts of protein were subjected to SDS-PAGE, followed by transfer onto PVDF membranes. Membranes were incubated with primary antibodies against ASPN, TGF-β1, and GAPDH, then with HRP-conjugated secondary antibodies. Signals were visualized using enhanced chemiluminescence (ECL) and analyzed with ImageJ software.

### Statistical analysis

Statistical analyses were conducted using R software (version 4.4.2). Differences between groups were evaluated using unpaired two-tailed Student’s t-tests. Correlation analyses were performed using either Pearson or Spearman correlation coefficients, depending on data distribution. A p-value less than 0.05 was regarded as statistically significant.

## 3 Results

### Screening of differentially expressed genes associated with CRL and HTS

**Figure 2:**
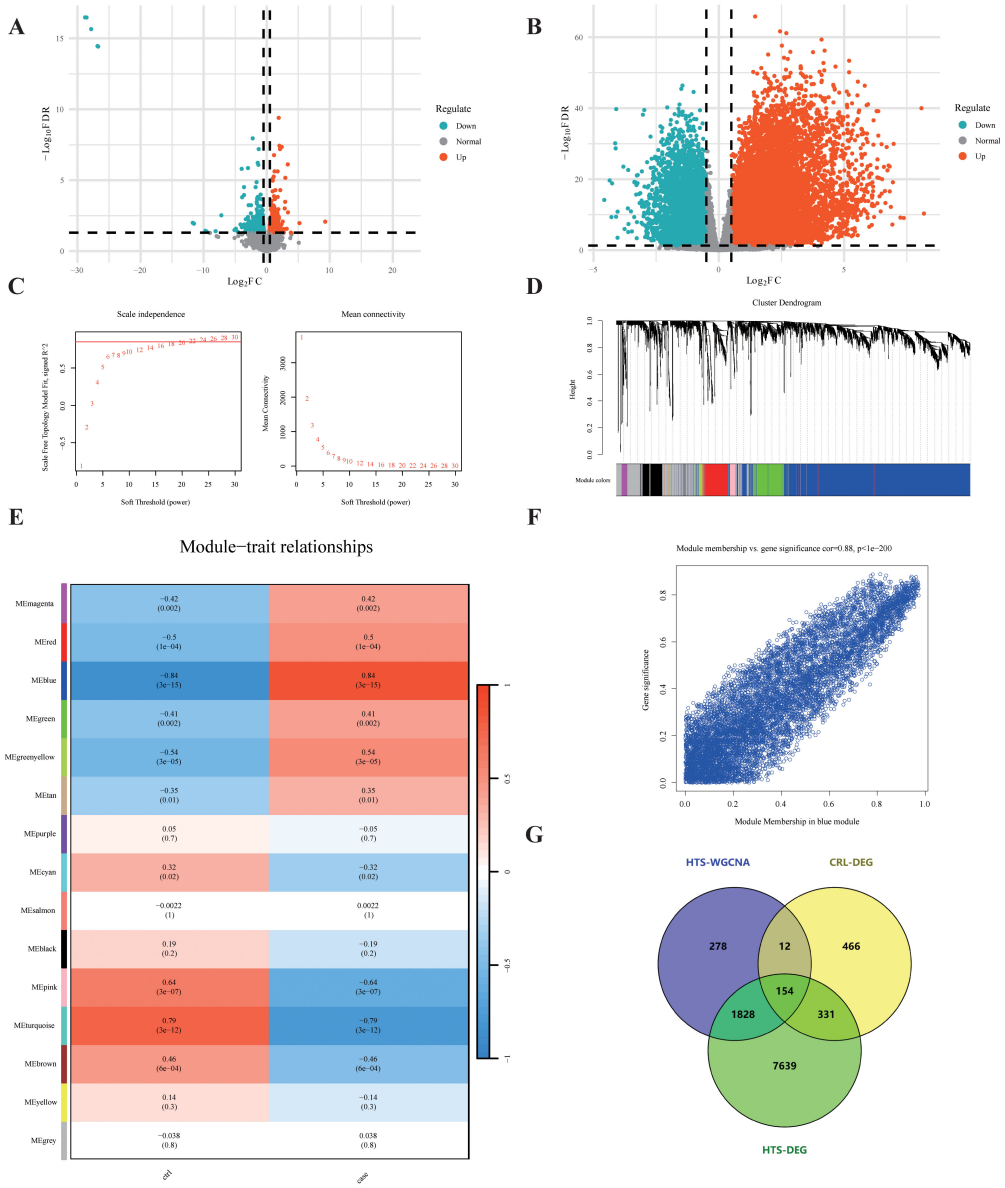
Shared gene screening. **(A, B)** Volcano plots illustrate the distribution of DEGs in CRL and HTS. **(C)** Network topology analysis determines the soft-threshold power for scale-free topology. **(D)** Dendrogram reveals gene clustering and module detection. **(E)** Module–trait correlation heatmap shows the association between each module and disease status, with red indicating positive and blue negative correlations. **(F)** Scatter plot depicts the correlation between module membership and gene significance within a significantly associated module. **(G)** Venn diagram identifies 154 shared genes overlapping across the HTS-WGCNA module, CRL-DEGs, and HTS-DEGs.

To investigate fibrosis-associated transcriptional changes in CRL and HTS, differential expression analysis was first performed. In the GSE255848 dataset (CRL), 963 DEGs were identified, comprising 613 downregulated and 350 upregulated genes (**Figures 2A, B**). Similarly, 9952 DEGs were obtained from the GSE178411 dataset (HTS), including 4390 downregulated and 5562 upregulated genes.

### Co-expression network analysis reveals HTS-associated gene modules

To further uncover disease-relevant gene modules in HTS, WGCNA was performed using the GSE178411 dataset. A soft-thresholding power of β = 22 was chosen to achieve a scale-free network topology (**Figure 2C**), resulting in the identification of 15 distinct gene co-expression modules (**Figure 2D**). Among these, the blue module exhibited the strongest association with HTS status (|r| = 0.84, p = 3e−15) and encompassed 2,272 genes (**Figures 2E, F**), which were selected for subsequent analyses.

### Identification of shared fibrosis-associated genes in CRL and HTS

We next identified overlapping fibrosis-related genes by comparing three sets: CRL DEGs (963 genes), HTS DEGs (9952 genes), and genes in the blue module from WGCNA (2272 genes). A total of 154 shared genes were identified using an online Venn diagram tool (**Figure 2G**), representing potential mediators of common fibrotic pathways in both conditions.

### PPI network construction and prioritization of candidate hub genes for functional relevance

**Figure 3:**
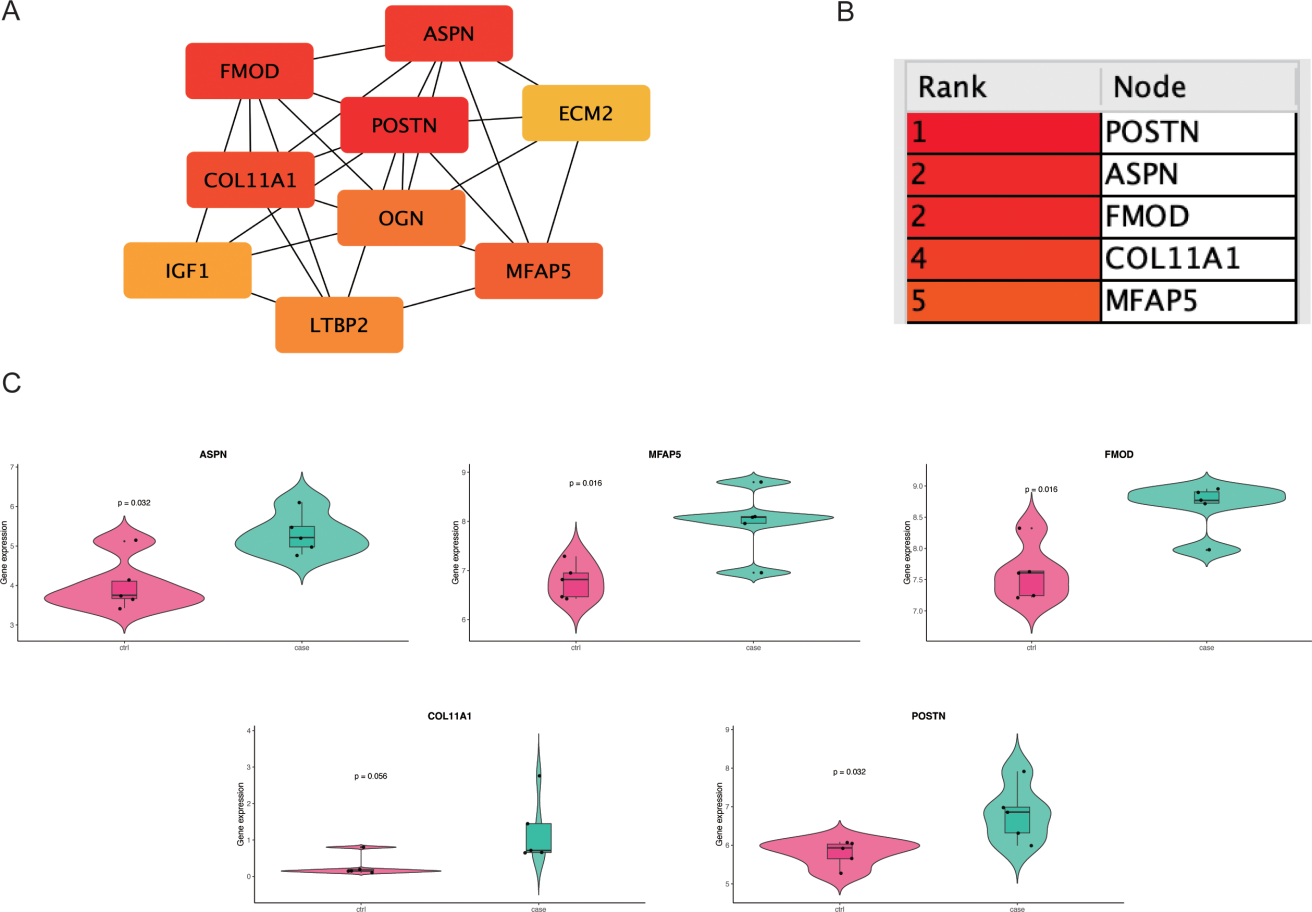
This figure demonstrates the identification and validation of fibrosis-related hub genes. **(A)** A protein–protein interaction (PPI) network constructed from DEGs reveals the connectivity among candidate genes. **(B)** The top 5 hub genes are ranked by degree centrality. **(C)** Violin plots compare ASPN, FMOD, POSTN and MFAP5 expression between CRL and control samples.

The 154 shared genes were input into the STRING database to generate a PPI network, which was visualized in Cytoscape (**Figure 3A**). Using the MCODE plugin, five highly interconnected hub genes were identified: POSTN, ASPN, FMOD, COL11A1, and MFAP5 (**Figure 3B**). Further expression analysis revealed that only four of these genes(POSTN, ASPN, FMOD, and MFAP5) exhibited statistically significant differential expression between lymphedema and control tissues (**Figure 3C**), indicating their potential biological relevance in the fibrosis pathogenesis of lymphedema.

### Exploratory ML identifies ASPN, FMOD, and MFAP5 as consistent fibrosis-associated genes

To further support hub gene prioritization, we applied RF and LASSO models to the five genes identified from the PPI network. LASSO identified ASPN, FMOD, and MFAP5 as candidate predictors in GSE255848. RF feature importance ranking confirmed these same three genes. Venn analysis showed complete overlap between both models. Although based on a limited dataset, this exploratory analysis yielded results consistent with the core PPI findings. Notably, ASPN showed the most robust upregulation in chronic lymphedema tissue. Functionally, ASPN modulates ECM remodeling and TGF-β signaling; FMOD is involved in collagen fibrillogenesis; and MFAP5 contributes to microfibril integrity. These findings are included in **Supplementary Figure S1**, and should be interpreted as supportive observations rather than conclusive evidence, due to the small sample size.

### Immune cell infiltration patterns and correlation with hub genes

**Figure 4:**
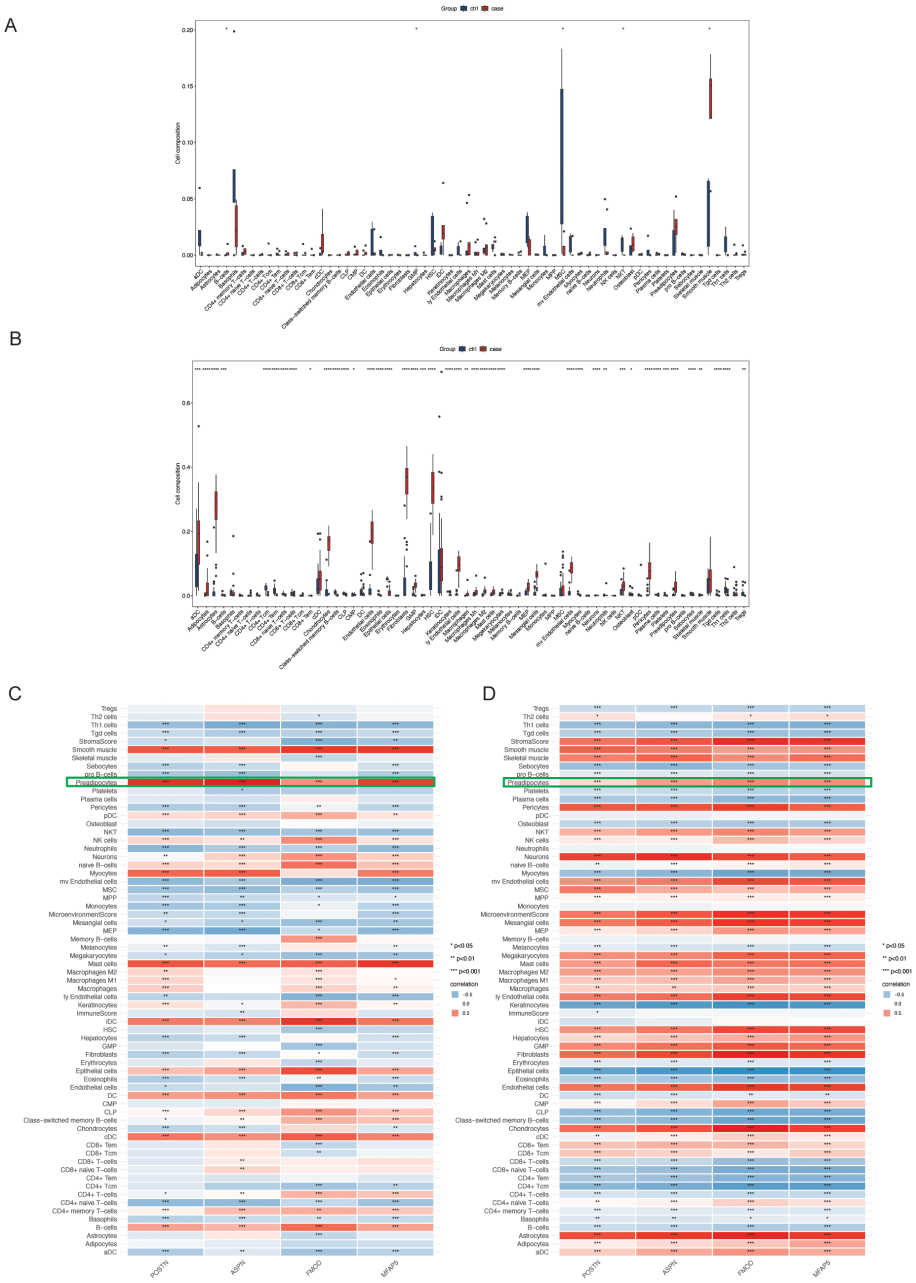
This figure illustrates the immune cell infiltration characteristics. **(A, B)** Box plots compare the relative abundance of immune cell subsets between CRL and HTS groups, inferred using the xCell algorithm; statistical significance is indicated by asterisks. **(C, D)** Heatmaps depict hierarchical clustering and estimated cell-type proportions across samples.

Immune microenvironment profiling via the xCell algorithm revealed distinct immune cell infiltration patterns between disease and control groups in both datasets (**Figures 4A, B**). Correlation analysis showed that POSTN, ASPN, FMOD, and MFAP5 were most strongly associated with preadipocyte infiltration in both CRL and HTS samples (**Figures 4C, D**), suggesting a link between mesenchymal transition and immune remodeling in fibrosis.

### ASPN identifies ADSCs associated with advanced fibrosis in CRL

**Figure 5:**
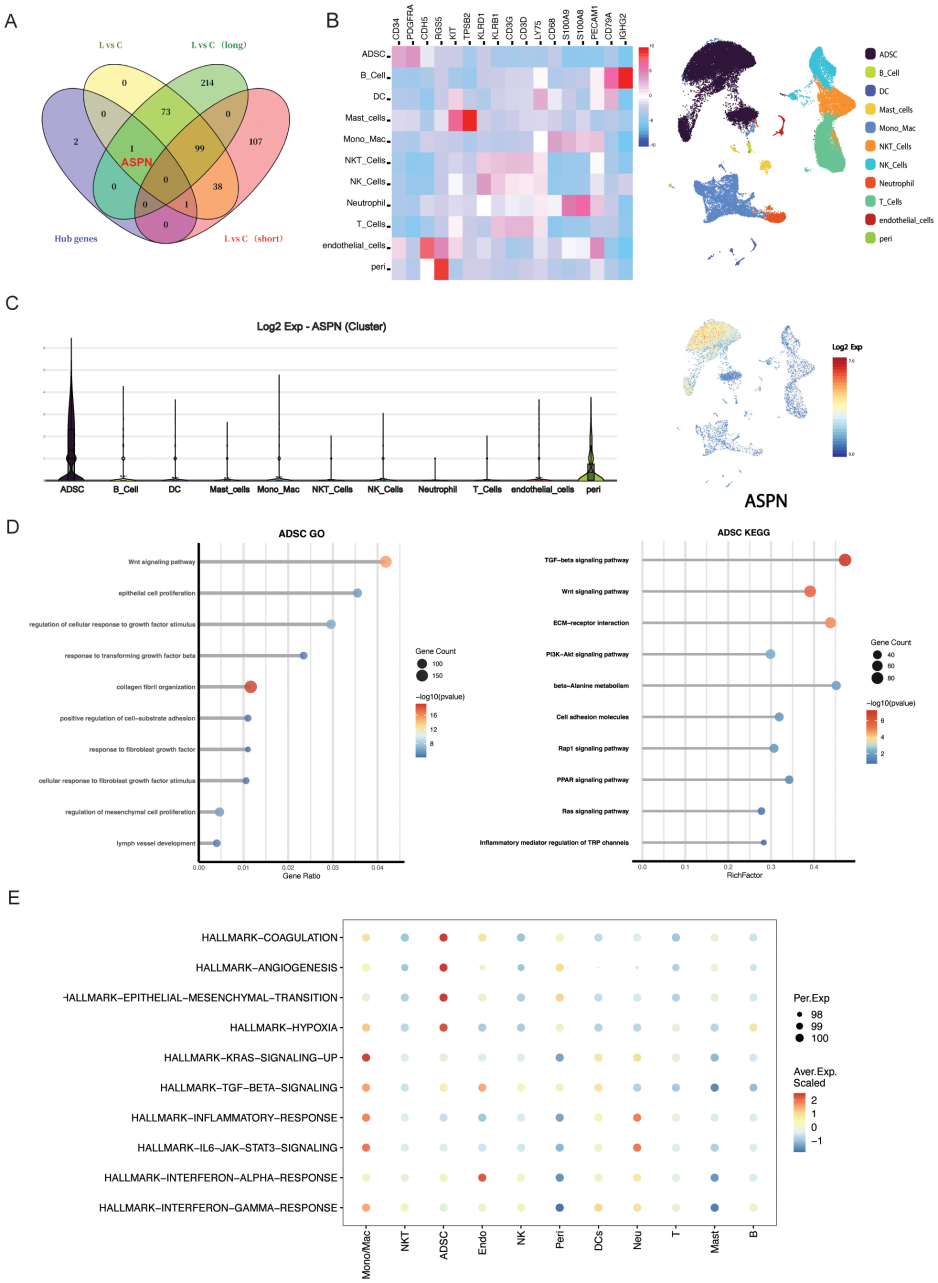
The expression characteristics and functional implications of ASPN at the single-cell transcriptomic level. **(A)**Venn diagram illustrating the overlap of key DEGs identified through multiple analytical approaches; notably, ASPN is among the DEGs in the long-duration group. **(B)** Heatmap displays expression levels of ASPN co-expressed genes across cell subsets, and UMAP plot shows their spatial distribution. **(C)** Violin plot indicates that ASPN is predominantly expressed in stromal cells. **(D)** GO and KEGG enrichment analyses show that ADSCs in CRL samples are significantly enriched in fibrosis-related pathways, including ECM remodeling, TGF-β signaling, and cell migration. **(E)** Hallmark analysis reveals activation of EMT, inflammation, and TGF-β pathways in ASPN-positive cells.

To assess the biomarker potential of candidate genes, we analyzed an independent bulk RNA-seq dataset from CRL patients stratified by disease duration (<3 years vs. >14 years). Among all candidates(POSTN, ASPN, FMOD, MFAP5), ASPN was the only gene consistently overlapping with differentially expressed genes (DEGs) in long-term cases across multiple comparisons (**Figure 5A**), indicating a strong association with advanced fibrotic stages. To elucidate the cellular context of ASPN expression, we conducted a focused analysis on scRNA-seq data from CRL tissue. ASPN expression was enriched in a specific subset of stromal cells (**Figures 5B, C**). Notably, ASPN expression was also observed in a subset of pericytes, suggesting that these cells may also participate in the fibrotic microenvironment in CRL. Violin plots further demonstrated that ASPN expression was largely confined to ADSCs, with negligible expression in immune and epithelial compartments (**Figure 5D**). GO and keg enrichment of ADSCs cells revealed associations with key fibrotic pathways, including Wnt signaling, TGF-β response, collagen fibril organization, and mesenchymal cell proliferation (**Figures 5E, F**). Additionally, AUCell hallmark gene set analysis showed that ADSCs were enriched for EMT, TGF-β, IL6–JAK–STAT3, and hypoxia signaling pathways (**Figure 5G**), underscoring their active role in extracellular matrix remodeling and inflammation-associated fibrosis. Together, these findings establish ASPN as a ADSCs-enriched marker of a distinct, pro-fibrotic stromal subpopulation, supporting its utility as both a biomarker and a potential therapeutic target in late-stage CRL fibrosis.

### ASPN expression correlates with fibrotic severity and disease progression in CRL

**Figure 6:**
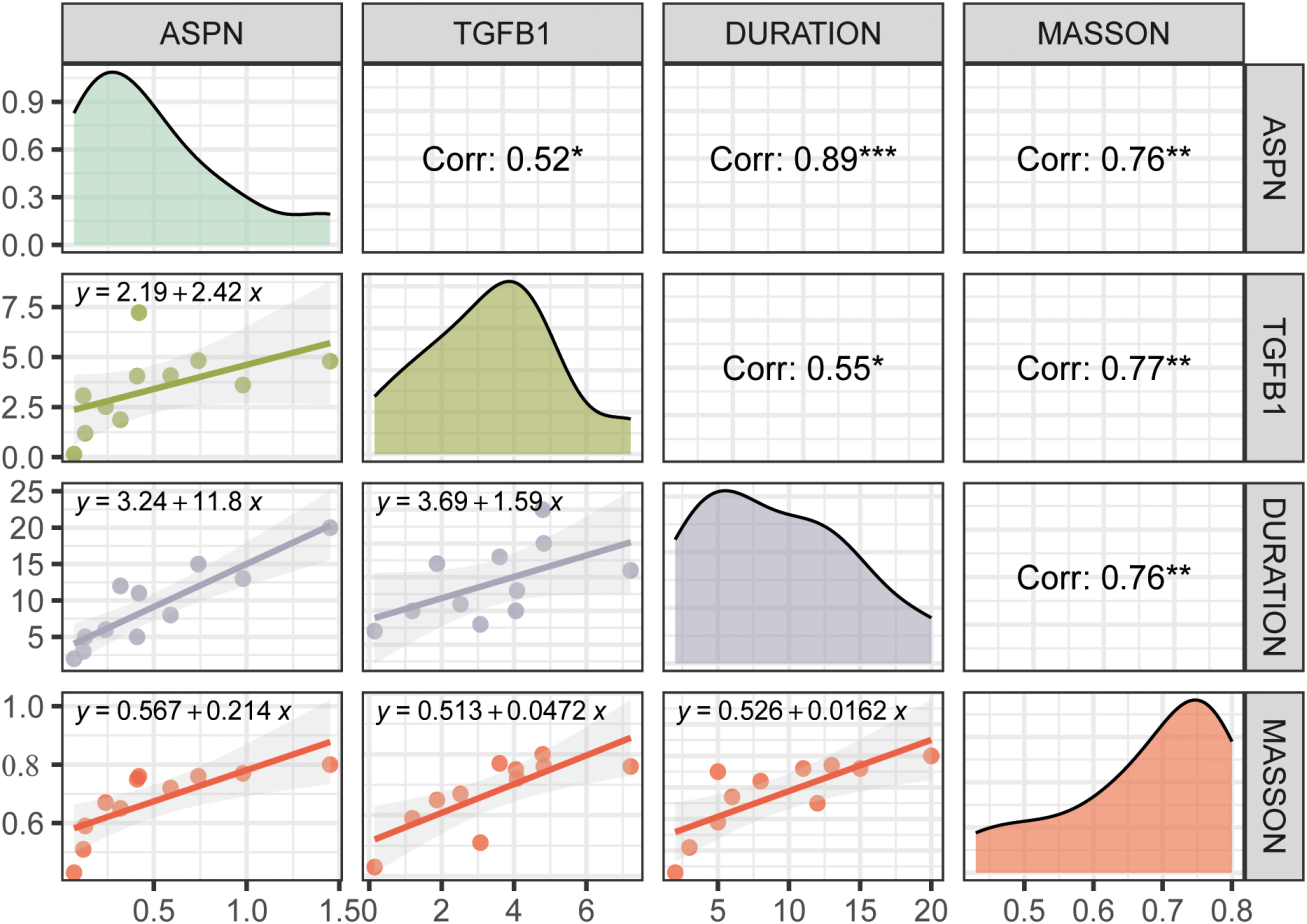
This figure presents correlation analyses among ASPN, TGFB1, disease duration, and Masson staining score. Diagonal panels show density distributions of each variable; the upper triangle displays Pearson correlation coefficients with significance levels indicated by asterisks (*p<0.05, **p<0.01, ***p<0.001); the lower triangle contains linear regression plots with fitted equations. ASPN expression is positively correlated with TGFB1, disease duration, and collagen deposition, suggesting its involvement in the fibrotic progression of CRL tissue.

To elucidate the role of ASPN in CRL fibrosis, we performed a combined histological and molecular analysis of skin tissue samples from 11 CRL patients with varying disease durations and degrees of fibrosis (**Supplementary Material 1**). Western blot analysis revealed a increase in ASPN protein expression correlated with longer disease duration, more severe histological fibrosis, and elevated TGF-β1 levels (**Figure 6**, **Supplementary Material 2**). Shapiro–Wilk tests confirmed normal distribution for all variables (Masson staining, disease duration, ASPN, and TGF-β1; all p > 0.05), supporting the use of Pearson correlation. Quantitative image analysis showed a positive correlation between collagen content and disease duration (r = 0.76, p = 0.0032), indicating progressive fibrotic remodeling over time. Importantly, expression levels of ASPN and TGF-β1 were positively correlated not only with disease duration (r = 0.89, p = 0.0001 and r = 0.55, p = 0.033, respectively) but also with the extent of collagen deposition observed in Masson staining (r = 0.76, p = 0.0035 and r = 0.77, p = 0.0026, respectively). In summary, these findings indicate that ASPN expression is associated with TGF-β1 levels and may have potential as a biomarker for disease progression and the severity of fibrosis.

### Inflammation and fibrosis are coordinated through ADSC–immune cell interactions

**Figure 7:**
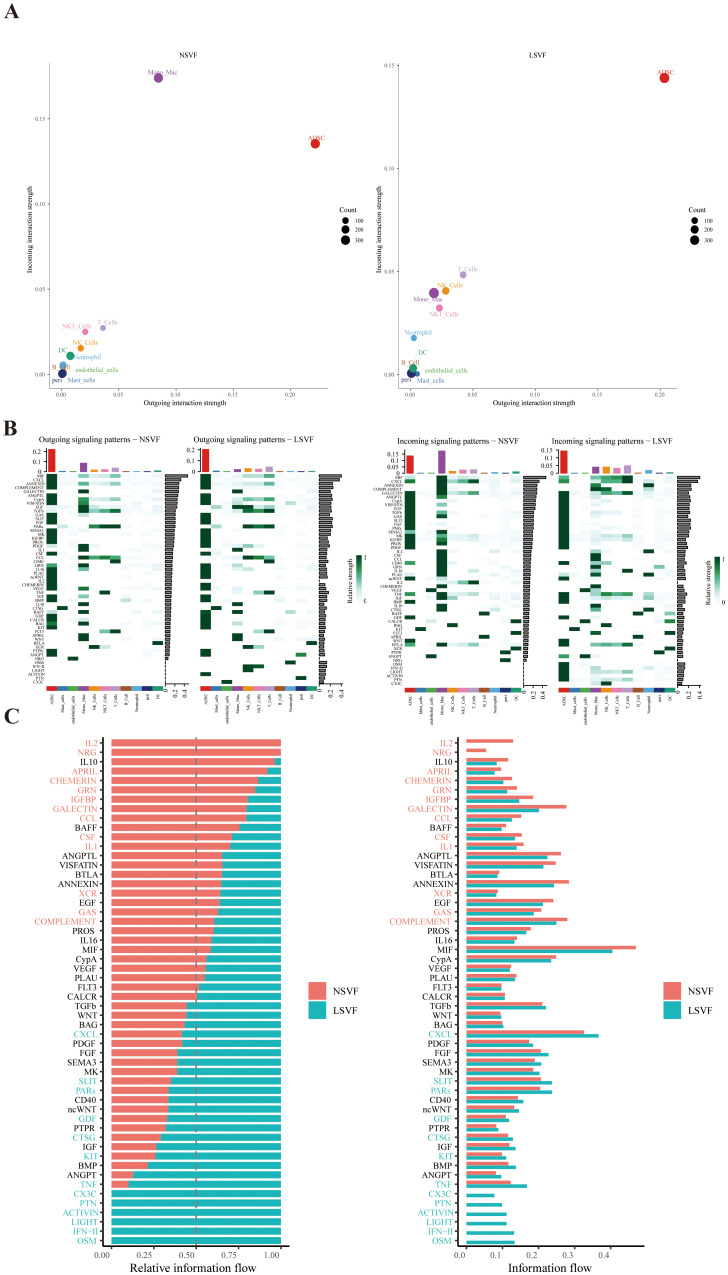
The figure illustrates the differences in intercellular communication signaling between NSVF and LSVF groups. **(A)** Outgoing and incoming information flows of various cell populations were quantified using CellChat. **(B)** Heatmaps show the pattern of outgoing and incoming signals across cell types in both groups. **(C)** Bar plots depict the relative importance and signal strength of specific signaling pathways in NSVF (red) and LSVF (blue).

CellChat analysis revealed enhanced intercellular communication in CRL tissues relative to controls (**Figure 7A**). Notably, TNF and IFN signaling pathways were upregulated and primarily derived from immune cell, with ADSCs acting as the main recipient cells (**Figures 7B, C**), suggesting that chronic inflammation may drive ADSC activation and downstream ECM remodeling. In the LSVF(lymphedema stromal vascular fraction) group, this communication network was markedly amplified. Especially, T cells exhibited increased outgoing signaling, while ADSCs received a significantly greater proportion of incoming signals compared to the NSVF(normal stromal vascular fraction) group. Several pro-inflammatory and pro-fibrotic pathways—including CXCL, OSM, IFN, and TNF—were notably enriched in LSVF, reflecting a shift toward sustained fibrotic activation. The elevated signaling from multiple immune subsets to ADSCs likely contributes to persistent ECM deposition and progressive tissue remodeling, characterizing advanced fibrotic stages.

## 4 Discussion

Chronic inflammatory processes in CRL accelerate tissue fibrosis and significantly diminish patients’ QOL, while also imposing considerable economic burden. As fibrosis advances, there remain limited therapeutic options for late-stage CRL (20). Although some pharmacological agents have shown promise in mitigating fibrosis associated with lymphedema, most are still in preclinical stages, making more invasive surgical excision the primary means of restoring limb function (21, 22). Although clinical examination and histopathological analysis can gauge the degree of CRL fibrosis to some extent, there is a lack of specific molecular biomarkers that reliably capture disease severity (23). Identifying fibrosis-specific markers is therefore critical for improving disease monitoring and tailoring therapeutic strategies.

Fibrosis in CRL is driven by multiple pro-inflammatory cytokines, but the precise downstream mechanisms and cellular interactions remain poorly understood (24, 25). In this work, we employed an integrated bioinformatics to investigate the potential shared fibrotic pathways between CRL and HTS. By analyzing two public transcriptomic datasets (GSE255848 and GSE178411), we identified 154 genes implicated in CRL fibrosis-related pathways. Further PPI analysis prioritized five key hub genes (POSTN, ASPN, FMOD, COL11A1, and MFAP5). During follow-up validation, COL11A1 was found not to be differentially expressed between lymphedema and normal tissues, and was therefore excluded from further analysis. Additionally, using an independent dataset, we observed that only ASPN was significantly upregulated in patients with long-term CRL (duration >14 years)—a pattern that aligns closely with the chronic progression of fibrosis in lymphedema. Additional immune infiltration and scRNA-seq analyses revealed that ADSCs are the predominant cellular source of ASPN in CRL tissues. Notably, ASPN expression was also detected in a subset of pericytes, suggesting that these cells may contribute to the fibrotic microenvironment in CRL alongside ADSCs and fibroblasts.

ASPN, a member of the small leucine-rich repeat protein (SLRP) family, modulates ECM formation and fibroblast activation in various fibrotic conditions, such as idiopathic pulmonary fibrosis, cardiac fibrosis, and HTS (26). Interestingly, some studies suggest ASPN can be antifibrotic under certain conditions—reflecting its potential dual nature across different disease settings (27–29). This duality underscores the need to clarify ASPN’s precise function in CRL’s unique inflammatory environment. Our findings support the notion that ASPN is closely tied to TGF-β signaling, a central pathway in fibrotic remodeling (30). In CRL tissues, heightened ASPN expression correlates strongly with both extended disease duration and increased histological fibrosis, indicating it may drive core processes of collagen deposition and tissue restructuring.

Moreover, functional enrichment analyses of ADSCs revealed upregulation of key fibrotic pathways, including Wnt signaling, TGF-β response, collagen fibril organization, and mesenchymal proliferation. Hallmark pathway activity further highlighted their involvement in EMT, IL6–JAK–STAT3, and hypoxia signaling, underscoring their active role in fibrotic remodeling and inflammation-associated fibrosis. These findings establish ASPN as a robust, ADSC-enriched marker of a pro-fibrotic stromal subset, supporting its potential use as both a biomarker and therapeutic target in advanced CRL fibrosis. To our knowledge, this is the first study to systematically investigate the transcriptomic overlap of fibrosis-associated genes between HTS and CRL, providing novel evidence for shared molecular signatures underlying fibrotic remodeling in these distinct but related conditions.

Importantly, CellChat analysis provided additional insights into the intercellular dynamics underlying fibrosis progression. Compared with controls, CRL tissues exhibited significantly enhanced cell–cell communication, particularly along TNF and IFN signaling axes originating from immune cells and targeting ADSCs. These interactions were further amplified in LSVF (late-stage, severe fibrosis) tissues, where T cells contributed to elevated outgoing signals. Concurrently, ADSCs received significantly more incoming communication, emphasizing their role as signal integrators within the fibrotic niche. Notably, several pro-inflammatory and pro-fibrotic pathways—including CXCL, OSM, IFN, and TNF—were upregulated in LSVF, indicating a shift toward sustained fibrotic activation and ECM deposition in advanced disease states.

Collectively, our findings highlight a multifaceted interaction between chronic inflammation and mesenchymal activation in CRL. ADSCs emerge as central effectors within this fibrotic circuit, receiving cues from diverse immune subsets and orchestrating downstream remodeling. These insights provide mechanistic evidence linking immune dysregulation with ADSCs activation in CRL and reinforce the concept that inflammation-driven stromal reprogramming underlies disease progression.

Clinically, ASPN holds promise as a biomarker for identifying early fibrotic changes and gauging fibrosis severity in CRL. Measuring ASPN levels may facilitate the early detection of high-risk patients, enabling timely intervention to halt or reverse irreversible fibrosis. Additionally, targeting ASPN-related pathways offers a potential avenue for novel therapeutic approaches against both CRL and HTS. Nonetheless, this study has certain limitations. First, our reliance on publicly available transcriptomic datasets may introduce batch effects and sample heterogeneity, potentially affecting reproducibility. The relatively small sample sizes and the limited number of CRL-focused datasets also constrain further verification of the identified hub genes in larger, more diverse populations. Although we applied WGCNA and ML methods to bolster reliability, further experimental validation across broader cohorts is necessary. Second, while scRNA-seq confirms that ADSCs are the primary source of ASPN in CRL tissues, the specific mechanisms by which ASPN mediates ECM remodeling in chronic inflammation remain incompletely defined. Although our findings point to a strong correlation between ASPN expression and the severity of fibrosis, in-depth *in vitro* and *in vivo* studies are needed to elucidate ASPN’s mechanistic contribution to CRL pathogenesis.

## Conclusion

In summary, this is the first study to integrate CRL and HTS data—utilizing both ML and bioinformatics—to delineate a common set of fibrosis-associated genes. By examining scRNA-seq and bulk RNA-seq data spanning different durations of CRL, we identified ASPN as a pivotal gene predominantly expressed in ADSCs and elevated in long-term CRL. Subsequent validation in patient samples further confirmed ASPN’s positive correlation with disease duration and histological fibrosis severity. These findings highlight ASPN as an essential biomarker for fibrotic progression in CRL and shed light on promising avenues for both diagnostic and therapeutic advancements.

## Data Availability

The datasets presented in this study can be found in online repositories. The names of the repository/repositories and accession numbers can be found in the article/**Supplementary Material**.

## Supplementary material

Supplementary Material 1 Sample information.

Supplementary Material 2 Western blot results.

Supplementary Figure 1 ML Identifies ASPN, FMOD, and MFAP5 as Consistent Fibrosis-Associated Genes. **(A)** LASSO regression selects key features with optimal lambda determined by cross-validation. **(B)** Random forest analysis identifies important genes based on variable importance scores. **(C)** Venn diagram shows three overlapping genes(ASPN, FMOD, MFAP5) from LASSO and RF.

## References

[1] Brown S Dayan JH Kataru RP Mehrara BJ. The vicious circle of stasis, inflammation, and fibrosis in lymphedema. Plast Reconstr Surg. (2023) 151:330e–41e. doi: 10.1097/PRS.0000000000009866, PMID: 36696336 PMC9881755

[2] Beesley V Janda M Eakin E Obermair A Battistutta D. Lymphedema after gynecological cancer treatment: Prevalence, correlates, and supportive care needs. Cancer. (2007) 109:2607–14. doi: 10.1002/cncr.22684, PMID: 17474128

[3] DiSipio T Rye S Newman B Hayes S. Incidence of unilateral arm lymphoedema after breast cancer: a systematic review and meta-analysis. Lancet Oncol. (2013) 14:500–15. doi: 10.1016/S1470-2045(13)70076-7, PMID: 23540561

[4] Rockson SG. Advances in lymphedema. Circ Res. (2021) 128:2003–16. doi: 10.1161/CIRCRESAHA.121.318307, PMID: 34110905

[5] Iyer D Jannaway M Yang Y P. Scallan J. Lymphatic valves and lymph flow in cancer-related lymphedema. Cancers. (2020) 12:2297. doi: 10.3390/cancers12082297, PMID: 32824219 PMC7464955

[6] Dayan JH Ly CL Kataru RP Mehrara BJ. Lymphedema: pathogenesis and novel therapies. Annu Rev Med. (2018) 69:263–76. doi: 10.1146/annurev-med-060116-022900, PMID: 28877002

[7] Lilja C Madsen CB Damsgaard TE Sørensen JA Thomsen JB. Surgical treatment algorithm for breast cancer lymphedema—a systematic review. Gland Surg. (2024) 13:722–48. doi: 10.21037/gs-23-503, PMID: 38845835 PMC11150198

[8] Bowman C Rockson SG. The role of inflammation in lymphedema: A narrative review of pathogenesis and opportunities for therapeutic intervention. Int J Mol Sci. (2024) 25:3907. doi: 10.3390/ijms25073907, PMID: 38612716 PMC11011271

[9] Nurlaila I Roh K Yeom CH Kang H Lee S. Acquired lymphedema: Molecular contributors and future directions for developing intervention strategies. Front Pharmacol. (2022) 13:873650. doi: 10.3389/fphar.2022.873650, PMID: 36386144 PMC9640931

[10] Sung C Wang S Hsu J Yu R Wong AK. Current understanding of pathological mechanisms of lymphedema. Adv Wound Care. (2022) 11:361–73. doi: 10.1089/wound.2021.0041, PMID: 34521256 PMC9051876

[11] Sumiya R Kageyama T Sakai H Tsukuura R Yamamoto T. Characteristic computed tomography findings in female patients with secondary genital lymphedema. Lymphat Res Biol. (2025) 23(3):lrb.2024.0006. doi: 10.1089/lrb.2024.0006, PMID: 39937577

[12] Lee SO Kim IK. Molecular pathophysiology of secondary lymphedema. Front Cell Dev Biol. (2024) 12:1363811. doi: 10.3389/fcell.2024.1363811, PMID: 39045461 PMC11264244

[13] Frech FS Hernandez L Urbonas R Zaken GA Dreyfuss I Nouri K. Hypertrophic scars and keloids: advances in treatment and review of established therapies. Am J Clin Dermatol. (2023) 24:225–45. doi: 10.1007/s40257-022-00744-6, PMID: 36662366

[14] Jeschke MG Wood FM Middelkoop E Bayat A Teot L Ogawa R. Scars. Nat Rev Dis Primer. (2023) 9:64. doi: 10.1038/s41572-023-00474-x, PMID: 37973792

[15] Liu X Yuan M Xiang Q Li Z Xu F Chen W. Single-cell RNA sequencing of subcutaneous adipose tissues identifies therapeutic targets for cancer-associated lymphedema. Cell Discov. (2022) 8:58. doi: 10.1038/s41421-022-00402-5, PMID: 35725971 PMC9209506

[16] Hsiao HY Liu JW Pappalardo M Cheng MH. The impacts of lymph on the adipogenesis of adipose-derived stem cells. Plast Reconstr Surg. (2022) 151(5):1005–15. doi: 10.1097/PRS.0000000000010082, PMID: 36534068

[17] Karaman S Lehti S Zhang C Taskinen MR Käkelä R Mardinoglu A. Multi-omics characterization of lymphedema-induced adipose tissue resulting from breast cancer-related surgery. FASEB J Off Publ Fed Am Soc Exp Biol. (2024) 38:e70097. doi: 10.1096/fj.202400498RR, PMID: 39394863 PMC11580717

[18] The Gene Ontology Consortium. The Gene Ontology Resource: 20 years and still GOing strong. Nucleic Acids Res. (2019) 47:D330–8. doi: 10.1093/nar/gky1055, PMID: 30395331 PMC6323945

[19] Kanehisa M. KEGG: kyoto encyclopedia of genes and genomes. Nucleic Acids Res. (2000) 28:27–30. doi: 10.1093/nar/28.1.27, PMID: 10592173 PMC102409

[20] Sanka SA Chryssofos S Anolik RA Sacks JM. Advances in surgical management of chronic lymphedema: current strategies and future directions. Med Oncol. (2025) 42:44. doi: 10.1007/s12032-024-02576-2, PMID: 39806245 PMC11729126

[21] Zurbuchen EA Yu N Salibian AA. Modern approaches to lymphatic surgery: a narrative review. Transl Breast Cancer Res. (2025) 6:6–6. doi: 10.21037/tbcr-24-49, PMID: 39980814 PMC11836749

[22] Wei M Wang L Liu X Deng Y Yang S Pan W. Metformin eliminates lymphedema in mice by alleviating inflammation and fibrosis: implications for human therapy. Plast Reconstr Surg. (2024) 154(6):1128e-1137e. doi: 10.1097/PRS.0000000000011363, PMID: 38391208 PMC11584190

[23] Karayi AK Basavaraj V Narahari SR Aggithaya MG Ryan TJ Pilankatta R. Human skin fibrosis: up-regulation of collagen type III gene transcription in the fibrotic skin nodules of lower limb lymphoedema. Trop Med Int Health TM IH. (2020) 25:319–27. doi: 10.1111/tmi.13359, PMID: 31816141

[24] Duhon BH Phan TT Taylor SL Crescenzi RL Rutkowski JM. Current mechanistic understandings of lymphedema and lipedema: tales of fluid, fat, and fibrosis. Int J Mol Sci. (2022) 23(12):6621. doi: 10.3390/ijms23126621, PMID: 35743063 PMC9223758

[25] Will PA Kilian K Bieback K Fricke F Berner JE Kneser U. Lymphedema-associated fibroblasts are related to fibrosis and stage progression in patients and a murine microsurgical model. Plast Reconstr Surg. (2024) 154:688e–700e. doi: 10.1097/PRS.0000000000011141, PMID: 37832143

[26] Fan R Yan X Zhang W. Relationship between asporin and extracellular matrix behavior: A literature review. Med (Baltimore). (2022) 101:e32490. doi: 10.1097/MD.0000000000032490, PMID: 36595867 PMC9794316

[27] Lo HJ Tsai CH Huang TW. Apoptosis-associated genetic mechanisms in the transition from rheumatoid arthritis to osteoporosis: A bioinformatics and functional analysis approach. APL Bioeng. (2024) 8:046107. doi: 10.1063/5.0233961, PMID: 39507523 PMC11540442

[28] Xu L Li Z Liu SY Xu SY Ni GX. Asporin and osteoarthritis. Osteoarthr Cartil. (2015) 23:933–9. doi: 10.1016/j.joca.2015.02.011, PMID: 25689697

[29] Liu L Yu H Long Y You Z Ogawa R Du Y. Asporin inhibits collagen matrix-mediated intercellular mechanocommunications between fibroblasts during keloid progression. FASEB J. (2021) 35(7):e21705. doi: 10.1096/fj.202100111R, PMID: 34105826

[30] Huang S Lai X Yang L Ye F Huang C Qiu Y. Asporin promotes TGF-β–induced lung myofibroblast differentiation by facilitating rab11-dependent recycling of TβRI. Am J Respir Cell Mol Biol. (2022) 66:158–70. doi: 10.1165/rcmb.2021-0257OC, PMID: 34705621

