# Supplementary figures and images for: Identification of novel hub genes and pathways predictive of fibrosis progression in cancer-related lymphedema through integrated multi-omics
Source: Front Immunol. 2025 Aug 13;16:1625972. doi: 10.3389/fimmu.2025.1625972 (PMC12380543; doi:10.3389/fimmu.2025.1625972)

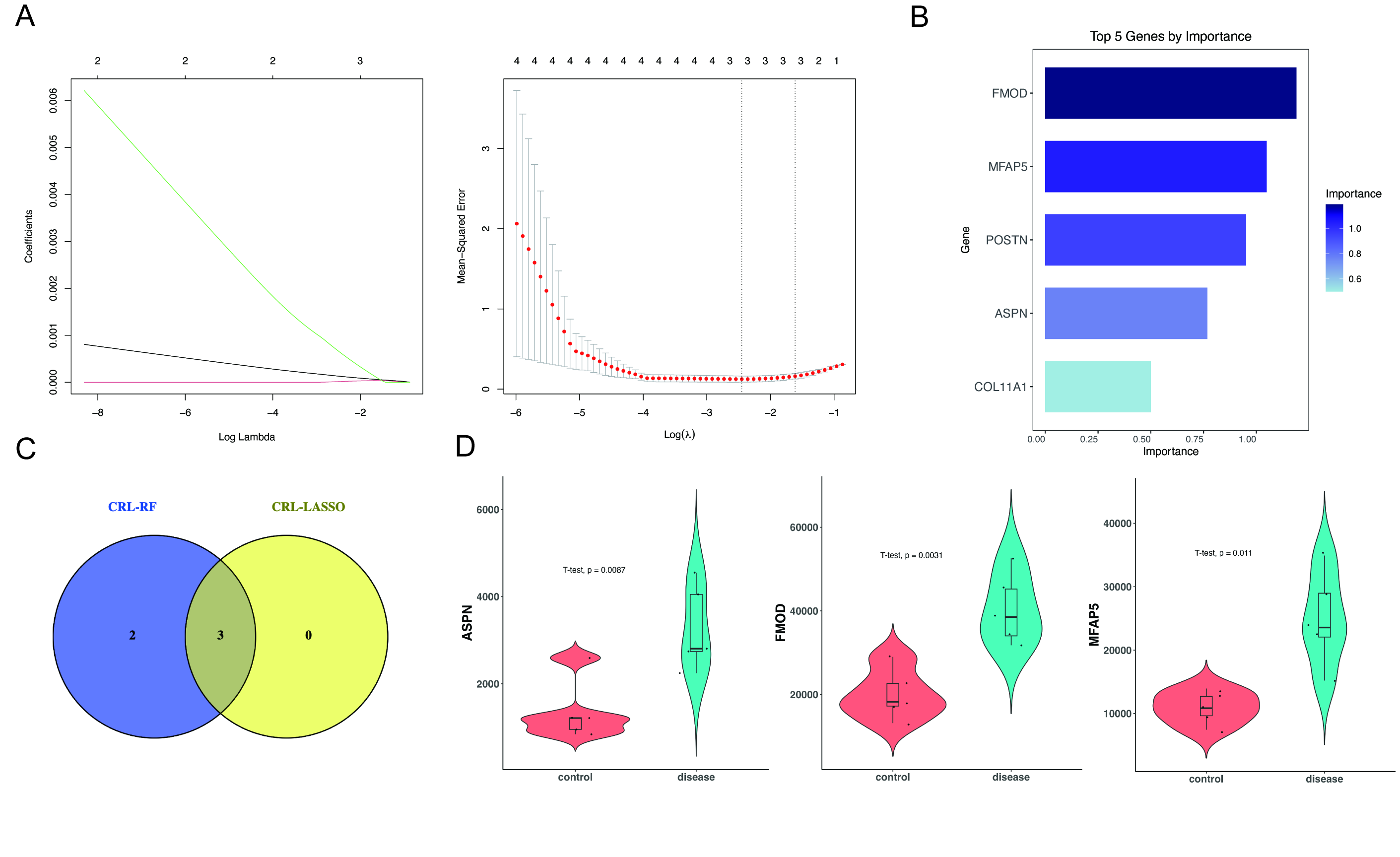

Supplement: Supplementary Figure 1 — ML Identifies ASPN, FMOD, and MFAP5 as Consistent Fibrosis-Associated Genes. (A) LASSO regression selects key features with optimal lambda determined by cross-validation. (B) Random forest analysis identifies important genes based on variable importance scores. (C) Venn diagram shows three overlapping genes(ASPN, FMOD, MFAP5) from LASSO and RF. [file Image1.tif]
